# Promoting opioids, a story about how to influence medical science and opinions

**DOI:** 10.3389/fmed.2024.1327939

**Published:** 2024-04-26

**Authors:** Maud Bernisson, Sergio Sismondo

**Affiliations:** ^1^Institute for Science in Society, Radboud University, Nijmegen, Netherlands; ^2^Department of Philosophy, Queen’s University, Kingston, ON, Canada

**Keywords:** opioids, pharmaceutical industry, marketing, ghost-management, Mallinckrodt, key opinion leader (KOL), medical education and communication company (MECC), epistemic corruption

## Abstract

Key origins of the opioid crisis in the US lie in some pharmaceutical companies’ substantial efforts to sell prescription painkillers. To legitimize opioids, the companies built up a body of medical science and opinions, and channels with which to communicate. Archival searches found 876 contracts that together provide information on how Mallinckrodt, an opioid manufacturer, attempted the ghost-management of medicine. These records—available because of litigation–involved contract research organizations, medical education and communication companies, publishers, professional societies, researchers, and other people who could be Mallinckrodt’s agents. Together, they produced and circulated scientific messages to increase physicians’ comfort with prescribing opioids. This article gives an overview of that activity, as seen in the contracts and related documents.

## Introduction

**Figure 1:**
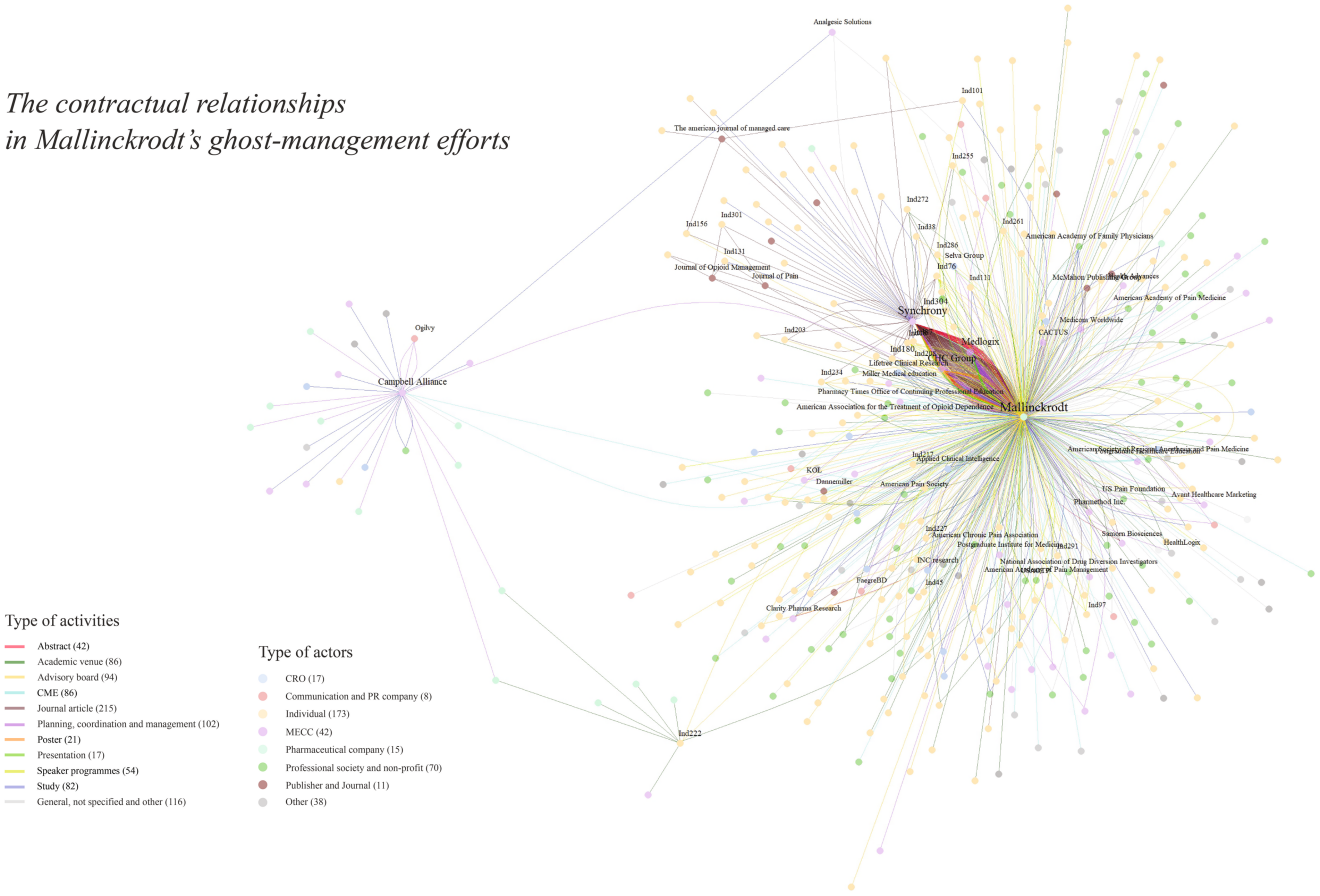
Contracts for the production or communication of medical science, from the Mallinckrodt Litigation Documents Archive (mapped with igraph 2.0.3).

What does the pharmaceutical industry’s influence on medical science and its communication look like? For one vantage point, see Figure 1, whose dots and lines trace a complex network of entities and people that fund and produce a small body of directed medical research, ghostwritten abstracts and articles, hired opinion leaders, and arrangements with publishers.

Pharmaceutical companies invest hundreds of millions of dollars to shape medical science, literature, and physicians’ opinions in target treatment areas (1–8). The contracts they maintain with their various partners—from individual doctors to multimillion-dollar consulting firms and medical publishers—spell out duties and expectations, payments, and procedures. These arrangements extend the companies’ reach and allow them to coordinate and supervise activities that support diverse marketing and influence strategies.

A number of pharmaceutical companies have been accused of contributing to the opioid crisis in the US (9, 10). The company Purdue has attracted the most public attention, and as a result smaller companies like Mallinckrodt have been mostly absent from public scrutiny (11). Mallinckrodt’s marketing through the structures of medicine and medical science has been central to lawsuits—and accusations that this marketing was often deceptive and unethical—but until now it has not been studied more generally.

Mallinckrodt is not a particularly well-known company, being much smaller than the industry’s giants. However, between the late 1990s and the mid-2010s, its extremely profitable generic drug business came to dominate the US prescription opioid market, with $18 billion in sales during that period (12). In 2010, the US Drug Enforcement Agency called Mallinckrodt “the kingpin within the drug cartel” of pharmaceutical companies selling opioids, especially with its popular oxycodone tablets known as “blues” (13). The company became a target of multiple lawsuits in the late 2010s, and many of them were settled between 2020 and 2022 via payments, bankruptcy, and restructuring. As a result of the litigation, the Industry Documents Archive acquired a trove of more than 1.4 million records that are accessible to the public. The availability of such documents provides unprecedented details for charting the activities of pharmaceutical companies (14). We present some cases and examples here.

## Methods

Working with the Mallinckrodt Litigation Documents Archive (15), we explored Mallinckrodt’s attempts to influence medical science and opinion. We found a number of contracts or formal agreements to provide services concerning the production or dissemination of medical science. Based on an initial informal survey of contract names, we built 17 keyword searches, and the archive turned up 3,862 documents. As Table 1 shows, most contracts are “consulting agreements,” “statements of work,” and “grants”—related to clinical trials, abstracts, publications, advisory boards, medical education and communication, and speaker programs about opioids. Some other documents, such as protocols, may sometimes function as contracts, but we set them aside for this study.

**Table 1:** Overview of the main types of contracts.

Type of contract	Description	# contracts
Statement of work	A statement of work describes services to be performed. In our data set they often involve medical education and communication companies (MECCs) or contract research organizations (CROs).	309
Consulting agreement	In our data set, most consultants were individuals hired for advisory boards, speaker programs, or roundtables.	245
Grant	In our data set, grants are mostly to non-profits, clinic/hospitals and professional societies. Some others are to MECCs (e.g., for continuing medical education courses).	170
Author agreement	In our data set, this type of agreement is usually made between a byline author and a MECC.	69
Reconciliation	A reconciliation is a document that includes all agreements, amendments, invoices and emails confirming the payments were authorized (usually by the pharmaceutical company).	36
Proposal	A proposal is a document listing the services and the associated narrative to convince a pharmaceutical company to engage in a contractual relationship with a subcontractor. Not all proposals are accepted.	20
Investigator-sponsored study agreement	These agreements stem from grants. A researcher is granted a certain amount of money by the pharmaceutical company to support a specific study.	18
Master services agreement	This is a general agreement where two companies agree on general contractual guidelines that will govern their relations for a specific amount of time, usually 1 year in this data set.	7
Authorship disclosure	This type of contract governs the contractual relationship between a byline author and the pharmaceutical company.	2

We manually removed duplicates and alternative versions of documents, and extracted 876 distinct and relevant contracts. We coded all contracts according to the activities related to the production of manuscripts and honorary authorship, publication planning, and other efforts to establish and communicate medical science (see Supplementary material for more information about the method). We refer to these activities as the “ghost-management” of medical knowledge.

Our dataset is a subset of the relevant contracts Mallinckrodt would have held. Most of the records we found in our searches were dated between 2011 and 2014, probably a result of the scope of the lawsuits. The archive does not contain most of the contracts originating with the central medical education and communication companies (MECCs) that Mallinckrodt hired to run many of its programs. Based on related documents, we conclude that a large amount of other relevant material was missed in the legal discovery process or was not deposited in the archive.

Starting from our database, we were able to search for other documents in the archive that followed from or contextualized the contracts. These allowed us to understand the activities of Mallinckrodt and its partners in more concrete terms. Categories of actors and of activities within ghost-management guided our analysis. Representative or clear cases are reported here.

Our focus on contracts offers a novel approach to studying corporate ghost management, one that we believe can be useful. Contracts establish responsibilities and actions. As such, they can provide a picture of pharmaceutical companies’ goals and the efforts they sponsor to achieve those goals.

## Results

### Contract research organizations and trials

Contract research organizations (CROs) specialize in the conduct of clinical trials. They may help pharmaceutical companies develop research protocols, or simply interpret and implement them. They recruit physicians to run sites and find subjects, or, especially for Phase I trials, may recruit subjects and run the trials themselves. They collect and audit data, and may do some analysis of it. Some CROs also offer regulatory and scientific writing. As other research has shown, pharmaceutical companies and CROs design and implement trials so as to maximize the chances of positive results (16–19). Like many pharmaceutical companies, Mallinckrodt generally outsourced its large clinical trials to CROs. These were chosen on the basis of competitive bids, the CROs’ expertise in running similar trials, and connections Mallinckrodt wanted to maintain (20). As for smaller trials, in our data set more than 30 trials were outsourced to individual researchers or small consortia working for clinics, hospitals, or even professional societies.

In our case, most contracted trials for new drug applications were somewhat standardized studies of efficacy and safety or addressed the FDA’s demand for risk evaluation and mitigation strategies for new opioids. That specific issue was also key to Mallinckrodt’s marketing plans, aligning with the company’s framing choices; it established unmet needs to be addressed.

The repeated phrase “unmet needs,” which is ubiquitous in the industry, spans medical and marketing opportunities, and so shapes research from the beginning. The particular unmet needs that Mallinckrodt sought to fill were bound up with its extended-release formulations. For example, a contract between the MECC Medlogix and Mallinckrodt, for the development of two manuscripts, provides a list of topics to address. They include “unmet needs in acute pain management,” which is an objective of an “unbranded” marketing strategy designed for the company’s drug Xartemis (oxycodone and acetaminophen [paracetamol]). The objective of this strategy is “[t]o further instill the unmet need in acute pain management and change the physician mindset with IR medication.” As a consequence, this argument would spread through several channels of scientific communications.

The company had identified that prescriptions by primary care physicians could be the source of the largest increase in the prescription opioid market. However, many primary care physicians were concerned about the possibility of dependence and abuse. These problems had created an epidemic in the previous decade because of the widespread availability of drugs sold by companies such as Purdue, Endo, and, of course, Mallinckrodt. Mallinckrodt planned to respond by marketing its new drugs, especially Exalgo XR (hydromorphone) and Xartemis XR, as intrinsically safer. The extended-release mechanism lessened pain more evenly over a 12-h period than did immediate-release pills, presumably providing patients and other users with less euphoria. In addition, the new pills were less amenable to being used recreationally. Such claims featured prominently in the company’s branding of its products.

Mallinckrodt also defined multiple laboratory studies and major clinical trials on human abuse liability—for example, by recruiting recreational drug users to compare the potential for abuse of the immediate- and extended-release forms of the pills. A 2014 document established in detail a protocol for a study of crushed extended-release (Xartemis) and immediate-release (such as Percocet) oxycodone/acetaminophen [paracetamol], administered intranasally. The subjects were to be “recreational, nondependent opioid users with intranasal experience.” The study was both to respond to the US Food and Drug Administration’s encouragement that manufacturers “develop opioid products with reduced abuse potential,” but the trial also showed that Mallinckrodt’s extended-release formulation had a lower level of “drug liking” (21) and was slower-acting, even when snorted, and so could be inferred to have less abuse potential (22). Such claims were made repeatedly in medical journal articles and in commercial medical media (23).

### Medical education and communication companies

MECCs offer services such as scientific medical marketing. They coordinate the production of manuscripts for submission to medical journals, abstracts and posters for conferences, and all manner of promotional material. They also organize continuing medical education courses, run advisory boards, and more. MECCs are central players, literally near the center of Figure 1.

Three MECCs dominate our data set: Synchrony, MedLogix, and the CHC Group. A few others are less prominent or are, in Figure 1, one link further from Mallinckrodt—contracting with sub-contractors.

The contracts with these MECCs typically describe deliverables, specify responsibilities, and set out due dates and costs. For example, a 2013 contract between Mallinckrodt and Synchrony was for six abstracts for the following year’s meeting of the American Pharmacists Association. Five of the six were revisions of previous versions and already had titles, and all six were in support of the forthcoming drug MNK 795, which would become Xartemis XR. Among its many responsibilities, Synchrony was expected to “liaise directly with client regarding the objectives …,” “provide literature research and analysis, identification and retrieval of appropriate references …,” “write abstract based on direction from authors using author-approved and client-supplied materials,” “facilitate client and author reviews,” and prepare the submission package. There were no authors listed for any of the abstracts and only small openings for authors to make contributions. This was clearly a project run by Synchrony, not by the eventual authors.

The contracts for journal articles are similar, though such articles tend to go through more extended and careful writing, review, and revision, making them more costly. Compared with other pharmaceutical companies, Mallinckrodt seems to have commissioned relatively few journal articles, probably fewer than 10 per year for its opioid franchises. This may be because, in the period covered by the archive, its main new products were extended-release versions of established and familiar combinations of opioids (hydromorphone or oxycodone and acetaminophen [paracetamol]). Its opioids were already selling at unbelievable rates—Mallinckrodt was selling nearly 40% of the prescription opioids in the US (24)—so the company did not need to build up a broad literature to establish a market.

Marketing activities are key in the early development of a manuscript. They might start with something like a lexicon workshop, establishing the key terms and phrases to be used. When we closely tracked the development of a single manuscript, we found nearly 200 documents in the archive, including dozens on marketing issues, more than a hundred manuscript drafts, and about 50 emails. The manuscript production process spanned a little more than a year.

During the manuscript development process, a project manager coordinates with the people who will become authors: “Dear Authors, Attached please find the first draft of the assessment of acute pain manuscript *Acute Pain Assessment: Assessing the Patient, Not Just the Pain*. While reviewing your section, please ensure both accuracy and flow. In addition, address any author queries noted in your section and include 5 acute pain assessment questions to include in the appendix.” The authors do not always respond even to these narrow requests, jeopardizing their status as authors and delaying the progress of the manuscript. If the authors do not answer, the MECC still has to fulfil its duties as specified in the contracts with the pharmaceutical company, and produce a suitable manuscript.

Publications support various key claims. The most important of these for Mallinckrodt, repeated over and over in publications sponsored by the company, was that pain was undertreated, and perhaps even underdiagnosed (25).

A more narrow theme was an emphasis on the abuse-deterrence of extended-release tablets, a theme common in documents we surveyed. For example, a 2011 proposal from the MECC Synchrony, for a publication plan for Mallinckrodt’s drug Exalgo, included at least one primary manuscript and two review manuscripts focused on abuse deterrence. One of the latter became an article published in the *Journal of Multidisciplinary Healthcare*, with the title “Update on prescription extended-release opioids and appropriate patient selection.” It provides a review of the pharmacokinetics of a wide variety of commercially available opioids, connecting them with patient populations (26). Although Exalgo is only one of a dozen products discussed in the article, email correspondence between Synchrony and Mallinckrodt identifies the article as “Exalgo publication by [author].”

Another key point was the importance of treating acute pain before it developed into chronic pain, a somewhat speculative phenomenon (27) that Mallinckrodt’s key opinion leaders (KOLs) and others call “chronification.” For example, in 2013 the MECC MedLogix agreed to produce a review article on the management of acute pain, and one of the claims would be: “Greater attention to patient and acute pain assessment and management leads to better patient outcomes including decreased chronification.” That project appears to have become a 2014 article in *Postgraduate Medicine*, with a cautious section on the “risk of chronification” (28). A number of other articles, both ones known to be sponsored by Mallinckrodt and ones by Mallinckrodt’s community of KOLs, refer to chronification in terms that imply, but generally do not state directly, that chronic pain is not just a normal trajectory but can be caused by the inadequate treatment of acute pain.

In all of these articles, the goal of pharmaceutical company publication seems to be to establish specific reference points for sales representatives to give assurances and reassurances to physicians (29, 30).

### Key opinion leaders and advisory boards

KOLs are researchers who are paid to represent companies’ interests, such as at sponsored events or academic venues (31). They often become authors of ghostwritten articles, present abstracts or posters and give talks at meetings large and small, speak at clinics, colloquia and special events, and serve on advisory boards (32). At the edges of Figure 1, most of the nodes radiating from the constellation of Mallinckrodt and the MECCs are KOLs. The pharmaceutical company and MECCs engage directly and indirectly with the KOLs. These agreements often offer a long-lasting relationship with the KOLs, who could become or strengthen their position as prominent experts on a selection of topics.

The term “advisory board” suggests that the pharma companies are seeking advice; in practice, pharmaceutical companies use the term to refer to a diverse array of things. The term most aptly describes assembled groups of physicians like a 2013 meeting of pain specialists held in Dallas, Texas. The detailed notes from that meeting make it clear that the organizers learned valuable lessons from the attendees and developed ideas for journal articles for physician education and more general talking points. The notes contain at least 12 rough concepts for articles. A few of these are for reports on data from trials and surveys, but most are for review articles on subjects ranging from “directions of acute pain management” to the more controversial “risk factors for pain chronification” and an article with the title “The Time Has Come: Pain Is a Disease.” Some of these ideas might turn into “proposed manuscript concepts,” complete with detailed outlines, target audiences, possible authors, and suggested journals. And some of these concepts would be expanded into actual manuscripts, ready to be submitted to medical journals.

A very different advisory board meeting in Orlando, Florida in 2014 focused on presentations of research opportunities to a small group of attendees apparently similar in composition to the Dallas group. The organizers’ notes clearly show that there was scant attention paid to gathering information from attendees; the few points recorded were clearly unfocused and not useful. This advisory board appears to have been organized to communicate information to the attendees and/or build or cement relationships, not to gather information.

Another model of advisory board meeting can be seen in another 2014 event in Orlando that brought together seven physicians’ assistants for orthopaedic surgeons, from US states where these assistants could legally write prescriptions. The meeting was held in a showy bar and seafood restaurant, part of a Disney resort. The agenda was focused on getting the attendees to identify which of two Mallinckrodt opioid painkillers was better for which patients in their practices, perhaps to encourage prescriptions. When companies want to bring physicians into their orbits, and when they want to make sales pitches, they often stage events aimed at creating positive feelings toward the company and its products.

### Speaker programs

Representing only a small number of items in our database, but a significant Mallinckrodt expenditure, were speaker programs. For example, a $677,110 contract with The Selva Group, another MECC, is for 175 speaker programs supporting Exalgo between October 1 and December 31, 2011. A “speaker program” is a single event in which, typically, a KOL gives a presentation to an assembled group of physicians. This might be an after-dinner talk or a lunchtime presentation in a clinic, and would typically focus on data and other evidence supporting the product. In the US, it is standard for these KOLs to be given zero flexibility in the content of their presentations because their talks are deemed “promotional” by the FDA, and so are indirectly regulated (33). An earlier contract had Selva revising four slide set modules for such programs, with approximately 40 slides per deck and speaker notes for all slides. These would be vetted by Mallinckrodt and a small number of KOLs chosen by Mallinckrodt, and Selva would incorporate any requested revisions.

The speakers are referred to as KOLs, though people in the companies involved make distinctions between “national level” KOLs and more local ones. A 2011 Exalgo speaker training event, also run by Selva and again in Orlando, included 70 attendees and their families from across the US. One organizer was concerned that an advisory board meeting in Tampa, Florida, scheduled for only a few days later, might lead some of the attendees to choose one event or the other. A Director at Mallinckrodt dismissed the concerns: Because the advisory board involved “national level” KOLs, only the guest presenter at the Orlando speaker event would be participating in both events.

Before they start delivering talks on behalf of a pharmaceutical company, most local KOLs are not influential physicians. Instead, it is the pharmaceutical companies’ hiring of them that makes them influential, transforming them into KOLs (33). They become networked with other physicians, and so become social nodes. In an important sense, then, pharmaceutical companies turn physicians into KOLs by providing them with training, resources, and venues to make these people influential.

### Continuing medical education

To keep their licenses, medical practitioners must complete accredited continuing medical education (CME) courses. The pitches MECCs make to pharmaceutical companies to design and run these courses are usually framed as “grant proposals” or “grant requests,” even when they are clearly linked to the company’s products and commercial interests (34).

One of the most expensive CME programs we found cost Mallinckrodt US$2.5 million. The MECC Global Education Group received this educational grant to organize sessions focusing on risk management in response to the FDA’s risk evaluation and mitigation plan. Informal goals listed by the CME manager ranged from “improve patient outcomes through education on higher doses,” and “[u]nderscore Mallinckrodt’s credibility with the FDA as a company that cares about … safe opioid prescribing” to “enhance[] Mallinckrodt’s reputation with Key Opinion Leaders (KOLs), patient advocacy groups and medical specialty societies involved in the program.” This CME program encompassed online workshops, virtual patient simulations, access to a platform that could evaluate physicians’ practices, online monographs, and scientific and editorial content development.

Publishers are also involved in CME programs. Publishing giant Elsevier has an “Office of Continuing Medical Education” that organizes and delivers CME courses. Elsevier’s claimed strength is its ability to advertise and recruit participants for its courses. In a proposal for a course for headache specialists and other neurologists, Elsevier, with partner AcademicCME, offered to run a live session at the 2013 meeting of the Southern Headache Society and then publish the audio and slides in the proceedings as an online CME course. That proceedings volume would be mailed to 9,000 specialists, but the event would also be promoted via Elsevier’s large email databases, via advertisements on websites, newsletters, electronic table of contents notifications, and a variety of major journals.

### Journals and publishers

The publishers are not incidental. Their business models can rely heavily on incomes from companies. For example, in 2015, the McMahon Publishing Group proposed to develop, in collaboration with KOLs, an article on one of a number of specific topics relevant to perioperative pain. It would appear in four of its trade publications, *Anesthesiology News*, *Pharmacy Practice News*, *General Surgery News,* and *Pain Medicine News*. In total, 128,500 copies would be printed and distributed to specialists. The agreed-upon price of this service was $198,250. McMahon had published multiple similar articles for Mallinckrodt over the previous few years.

Publishers benefit variously from their collaborations with pharmaceutical companies. For example, in 2015 the publisher Informa sent a proposal to Mallinckrodt with discounts for preprints, and perpetual open access. In exchange, Informa required Mallinckrodt “to publish 15 manuscripts in [its] core journals: *Postgraduate Medicine, Hospital Practice, The Physician and Sportsmedicine, Current Medical Research & Opinion*, and the *Journal of Medical Economics*.” Mallinckrodt had already purchased a license with *Postgraduate Medicine,* which meant that its submissions would receive expedited service and individual articles would not be subject to page fees.

*Postgraduate Medicine*, for its part, was actively trying to convince Mallinckrodt to submit articles and to purchase reprints. In 2014, in reference to a group of articles, an employee of the journal wrote: “Mallinckrodt’s attached articles are well over hundreds of views.” And in a follow-up email: “I can have 40,000 copies … in a couple of weeks, shrink wrapped in whatever increments you choose.” Tellingly, the subject line in the first of the email threads was “Mallinckrodt Postgraduate Medicine Articles,” not identifying the articles by authors: To the journal, the company’s role in their production was transparent.

## Discussion

Our searches of the Mallinckrodt Litigation Documents Archive provide some close-up views of a pharmaceutical company at work to influence medicine.

Our and others’ research has shown that one key to pharmaceutical companies’ success is their ability to shape the knowledge of prescribers. For its two new drugs, Xartemis and Exalgo, Mallinckrodt subcontracted to MECCs and CROs to deploy a marketing strategy whose key arguments were the need to treat pain, and the increased safety of extended-release opioids over immediate-release opioids. This strategy specifically highlighted existing concepts like chronification or pseudo-addiction to convince health-care practitioners to prescribe more opioids during an opioid crisis. Purdue had also used the term and idea of “pseudo-addiction” as part of its marketing strategy to boost OxyContin sales (35).

Arguments, often based on such concepts, were spread through channels like advisory boards, speaker programs, in the scientific literature or in specialized news outlets (30). KOLs could also take that information to physicians’ clinics and to CME courses, helping to make it the relevant information on which to focus. Sales representatives could take that information, sometimes in the form of reprints, into physicians’ offices.

Outsourcing with close supervision and coordination is the norm for pharmaceutical companies. Their positions as contractors provides them with leverage over their subordinate partners, allowing them to directly dictate their partners’ actions. For example, they typically hire MECCs through yearly contracts, and temporary missions are defined in more details through additional contracts. Such contingent arrangements make the MECCs dependent on the pharmaceutical companies. Documents from MECCs, such as proposals for publication plans, clearly align the work to the interests of the companies.

Individuals experience a different type of bond with the pharmaceutical companies. Again, the companies’ hiring of KOLs for particular tasks allows those companies to dictate actions to shape medical science and opinion. In addition, though, we observed that a number of KOLs had several relationships with Mallinckrodt, which would have strengthened conflicts of interest. Agreements between KOLs and the company define the terms of engagement but also build relationships. Thus, for example, authorship invitations can be considered rewards as can other invitations, even paying ones such as serving on advisory boards or participating in speaker bureaus.

Multiple CROs, MECCs, KOLs and publishers are eager to profit by implementing pharmaceutical companies’ strategies. In return, those companies can strengthen their bonds with actors they choose, who play by their rules, and who serve their interests. The relationships they maintain are asymmetric, allowing the pharmaceutical companies to establish the forms of those relationships and the shapes of their products. To the extent that pharmaceutical companies can control their intermediaries’ interventions into the stages of research and communication, they increase their position of dominance in the field. They also establish a huge and growing market for private players in the ghost management of medicine.

In the context of the opioid crisis, pushing opioid prescriptions has had dramatic consequences. According to the US Centers for Disease Control and Prevention, between 1999 and 2021, 280,000 people died from a prescription opioid overdose, and the number of deaths per year increased five-fold over that period (36). The epidemic of prescription opioid addiction preceded a one of non-prescription opioids, from which many more died. The crisis had shortened overall life expectancy by some months (37). According to one Mallinckrodt KOL, it did not (38). Hundreds of lawsuits have successfully argued that specific pharmaceutical companies, including Mallinckrodt, have been actively responsible for this crisis.

Mallinckrodt is a relatively minor player in the pharmaceutical industry, other than its outsized presence in the generic opioids business. We are not here arguing that it was a particularly skilled or strategic player, though gaps in the documentary record make it difficult to establish definite claims on this issue. Nonetheless, Mallinckrodt used the same tools and strategies as seen in the rest of the pharmaceutical industry. Perhaps, had it not played a significant role in a societal crisis, the company’s actions would not have attracted legal attention. But that attention allows us a detailed window into this company’s attempts to create channels of influence.

## Data availability statement

The original contributions presented in the study are included in the article/Supplementary material, further inquiries can be directed to the corresponding author.

## Ethics statement

Written informed consent was not obtained from the individual(s) for the publication of any potentially identifiable images or data included in this article because information was in the public domain.

## Author contributions

MB: Conceptualization, Writing – original draft, Writing – review & editing, Data curation, Methodology, Investigation, Analysis. SS: Conceptualization, Writing – original draft, Writing – review & editing, Investigation, Analysis.
