# Supplementary material for: Promoting opioids, a story about how to influence medical science and opinions
Source: Front Med (Lausanne). 2024 Apr 26;11:1327939. doi: 10.3389/fmed.2024.1327939 (PMC11082278; doi:10.3389/fmed.2024.1327939)
Supplement: Supplementary file 1 [file Data_Sheet_1.PDF]

Supplementary material

Maud Bernisson[1]
Sergio Sismondo[1, 2]

[1] Institute for Science in Society, Radboud University, Nijmegen, Netherlands,
[2] Department of Philosophy, Queen's University, Kingston, Canada

Materials and Methods

The Archive

The documents in the Industry Documents Library come from Mallinckrodt litigations (US Bankruptcy Court for the District of Delaware) and were made public following subpoenas and information requests from several Attorney general and civil investigative demands. The archive concerning Mallinckrodt includes documents from 2002 to 2020 and encompasses 1 398 993 documents (see Caleb Alexander et al., 2022).[1] These documents were provided by the US Courts to the UCSF industry documents library, hence their public availability. This archive is made of diverse documents, e.g., invoices, presentations, or e-mails, that concern marketing and sales. It also includes contracts, proposals, and invoices, which we retrieved to document Mallinckrodt's implementation of ghost management through its contractual relationships with a diverse range of stakeholders.

Search queries summary

Search queries were built departing from the type of contracts, which are template-based. For example, "author agreement." Nevertheless, a strong limitation was discovering enough different templates to be able to find more different templates. Consequently, our search strategy was to also use very broad queries, e.g., *"statement of work" "manuscript".*

Supplementary Table 1 Search queries and results

	Queries	Date of the query	#Result
**1**	"author agreement"	2022_11_28	437
**2**	"statement of work" "manuscript"	2022_11_28	819
**3**	reconciliation "working title"	2022_12_15	549
**4**	“INVESTIGATOR - SPONSORED STUDY AGREEMENT (ISS)”	2022_12_15	81
**5**	“research agreement”	2022_12_15	393
**6**	“Research organization master services agreement”	2022_12_15	12
**7**	“Research grant agreement”	2022_12_15	19
**8**	medlogix agreement	2023_01_11	984
**9**	Journal Publishing Agreement -"Subject: " -"Jurnista" -"ppt" -powerpoint -"Dahm et al Clin J Pain 1998.txt"	2023_01_24	154
**10**	grant compliance committee approval publish* -"Subject: " -"Jurnista" -"ppt" -powerpoint -"Dahm et al Clin J Pain 1998.txt"	2023_01_24	160
**11**	consulting agreement "Consultant possesses expertise in the area" → corrected to [consulting agreement "possesses expertise"]	2023_01_24	257
**12**	"statement of work" publication -ppt -"FW: " -"Cc: "	2023_03_06	1187
**13**	#11 corrected to [consulting agreement "possesses expertise"]	2023_03_06	328
**14**	"Mallinckrodt" consulting agreement speaker -ppt -"FW: " -"Cc: " -"daily news report"	2023_04_26	1,127
**15**	clinical trial* "statement of work" -ppt -"FW: " -"Cc: " -"daily news report"	2023_05_01	302
**16**	Mallinckrodt "statement of work" "speaker program" -ppt -"FW: " -"Cc: " -"daily news report"	2023_05_01	34
**17**	Grant Compliance Committee "Request for Funding"	2023_05_02	265

In addition to contracts, we found key documents like emails and proposals through serendipity. We did not search for these types of documents although they appeared to provide key information.

We removed from the selection all documents that had the same ID. We were left with 3,862 documents. We went manually through these documents and removed duplicates and alternative versions of documents (1305 out of 3862). Among all versions of a document, we privileged the oldest one, which usually includes the signatures of the parties involved in the contract. The final number of documents selected amounts to 876.

Overview of the data

Supplementary Table 2 Overview of the main types of contracts

Consultant	Client	Type of contract	#Contracts
**MECC**	Mallinckrodt	Statement of work	310
**Honorary author, researcher, MD, PharmD**	Mallinckrodt	Consulting agreement	245
**Institution, individual**	Mallinckrodt	Grant	170
**Honorary author**	MECC	Author agreements	69
**Honorary author, researcher, MD, PharmD**	Mallinckrodt	Reconciliation	36
**Institution**	Mallinckrodt	Proposal	20
**Researcher (PI)**	Mallinckrodt	Investigator-sponsored study agreement	18

**MECC, com or PR agency**	Mallinckrodt	Master services agreement	7
**Honorary author**	Editor/Publisher	Authorship disclosure	2

Based on the services they offer, categories of companies and actors overlap and different names can be used for the same or a different category.

Types of contracts

Authorship disclosure was almost absent from the final data set, even though they seem to be a common document. In 2016, Mallinckrodt drew its publications policy (qqdj0253), which requires that "Authors engaged in publication of our primary data must sign an Authorship Disclosure (Attachment 1), which incorporates by reference, any signed confidential disclosure agreement (CDA)" (qqdj0253, p4). The template of an authorship disclosure is part of the appendices of Mallinckrodt publications policy (qqdj0253, p10).
Master service agreements are very few in the data set. They are general agreements between the service provider and Mallinckrodt. They state the general rules for future contracts between both parties.
Statements of work (SOW) define the services to be provided and detail the costs and the timeline. Amendments to a statement of work can modify SOWs.
Amendments also exist for other types of contracts, like consulting agreements. Consultants who have already been working for Mallinckrodt can have their first agreement amended for another service.
Reconciliations are extensive documents that include fine-grained details of the costs (line by line), the SOW or agreement, sometimes e-mails are also included, and a few other documents that track back the history of the service and its costs. In the data set, we favoured reconciliation to SOW (we kept reconciliation instead of SOW when they referred to the same service).
Consulting agreements are contracts between individuals or companies and Mallinckrodt. They detail the service, the dates of the service and the cost. They also state specific rules that both parties should respect, e.g., in case of problems to deliver the service or intellectual property clauses (like in the Master Service agreements).
Author agreements are documents between MECCs (common) or Mallinckrodt (rare) and the honorary author. It provides details on the manuscript to be written, the author (and potential co-authors) and the rules related to authorship (e.g., ICMJE). It also distributes responsibilities between the author, the MECC and the sponsor.
Grants are a loose category. It includes all documents related to grant proposals and grant acceptances. It thus concerns a variety of actors. It also includes different types of grants: sponsorship, charitable contribution, unrestricted educational grants, or continuing education (lxjj0253, p1). As suggested by the type of grants listed, grants include different types of activities: continuous education (CME), and medical marketing (like publications).
Proposals concern all types of documents that offer detailed services with a proposed budget. Very different ones exist. For example, a proposal can offer a detailed plan to "Assess impact with prescribers and payers of new labelling requirements for ER/LA opioids on MNK-795" (xxxx0249). A specific type of proposal is **investigator-sponsored study agreements,** for which an independent researcher proposes a study to Mallinckrodt. It is an extensive document (often more than 100 pages), which includes extensive information on the study to be conducted, the author(s), the timeline and the fees.
In addition to these categories, a few other documents like emails were included to provide elements of context.

The figure (Figure 1)

In Figure 1, there are 374 nodes, that is, different actors involved in the network. While the data set contains 876 contracts, the figure shows 915 edges. This is because one sub-contractors might be many. If one contract mentions several clients and describes the budget of all clients, then, there is one edge per relationship among each of the clients (e.g., yjfc0254 describes services and costs per pharmaceutical company, then, edge 1 will represent pharma company 1 linked to the service contractor, edge 2 will represent pharma company 2 linked to the service contractor, etc.)

CROs and MECCs often offer similar activities (e.g., medical writing). Thus, the category “CRO” includes companies that offer to produce studies for Mallinckrodt. Companies that offer medical writing and other marketing services are categorized as MECCs.
We coded Covidien as Mallinckrodt. We also coded individuals working for Mallinckrodt and who have had an author agreement with Synchrony, as Mallinckrodt.
Finally, edges do not represent the number of activities per contract, only the amount of relationships between different actors. One contract can mention several activities, though. For example, an outlier specifies 145 speaker programs (e.g., lfvw0243). However, it is quite common to find in contracts several posters or abstracts (2 to 16). However, if a document contains several contracts, the different contracts were listed in the final data set (e.g., xnyw0232).

Limitations of the data

The number of documents included in the data set does not represent the overall publications by Mallinckrodt. Other documents provide information on many more publications (NSAIDs included), however, the corresponding contracts did not appear in the SERPs.
The dates of the documents show limitations to the data set:

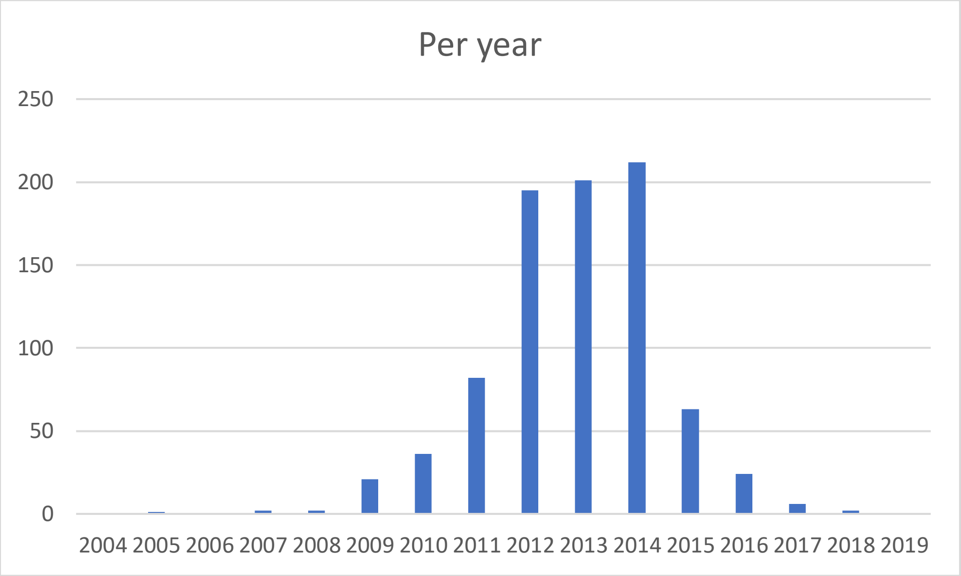

Supplementary Figure 1 Number of contracts per year

Other limitations concerned OCR, which is problematic to retrieve all documents relating to a search query. In addition, the librarians are working regularly on the database, which means that results might change over time. Finally, the search was also limited to the documents that came to our knowledge. There might be documents that can be retrieved with specific keywords that we were not aware of.

Anonymization

All individuals were anonymised on the figure. The results and the analysis are not altered by the anonymization, since we focused on the ghost management system rather than individuals.

References

[1] Alexander GC, Mix LA, Choudhury A, Taketa R, Tomori C, Mooghali M, Fan A, Mars A, Ciccarone D, Patton M, Apollonio DE, Schmidt L, Steinman MA, Greene J, Knight KR, Ling PM, Seymour AK, Glantz S, and Tasker K. 2022: The Opioid Industry Documents Archive: A Living Digital Repository American Journal of Public Health 112, 1126_1129, https://doi.org/10.2105/AJPH.2022.306951
